# Oncological Feasibility of Limited Neck Dissection in cN0 Supraglottic Laryngeal Cancer

**DOI:** 10.1002/hed.28081

**Published:** 2025-01-23

**Authors:** Sara Bassani, Giacomo Papi, Filippo Marani, Riccardo Nocini, Valentina Campagnari, Filippo Marchi, Marco Lionello, Chiara Varago, Alberto Paderno, Erika Crosetti, Francesco Mattioli, Edoardo Serafini, Matteo Fermi, Alessandro Rosti, Gerardo Petruzzi, Flaminia Campo, Elisa Bellini, Alberto Vito Marcuzzo, Chiara Angela Mineo, Giuseppe Mercante, Armando De Virgilio, Leone Giordano, Andrea Galli, Giuditta Mannelli, Luca Mazzetti, Gaetano Paludetti, Giorgia Rossi, Karol Zeleník, Daniel Marin Ramos, Rogèrio Aparecido Dedivitis, Giovanni Succo, Giancarlo Tirelli, Mario Bussi, Raul Pellini, Jacopo Galli, Giuseppe Spriano, Daniele Monzani, Giorgio Peretti, Andy Bertolin, Cesare Piazza, Daniele Marchioni, Livio Presutti, Gabriele Molteni

**Affiliations:** ^1^ Otolaryngology‐Head and Neck Surgery Department University of Verona Verona Italy; ^2^ Azienda Ospedaliera Universitaria Integrata of Verona Verona Italy; ^3^ Unit of Otorhinolaryngology—Head and Neck Surgery IRCCS Ospedale Policlinico San Martino Genoa Italy; ^4^ Department of Surgical Sciences and Integrated Diagnostics (DISC) University of Genova Genoa Italy; ^5^ Unit of Otorhinolaryngology Vittorio Veneto Hospital Vittorio Veneto Italy; ^6^ Department of Biomedical Sciences Humanitas University Milan Italy; ^7^ IRCCS Humanitas Research Hospital Milan Italy; ^8^ ENT Department University of Turin, Head and Neck Cancer Unit, San Giovanni Bosco Hospital Turin Italy; ^9^ Department of Otolaryngology Head and Neck Surgery University Hospital of Modena Modena Italy; ^10^ Department of Medical and Surgical Sciences, Alma Mater Studiorum University of Bologna Bologna Italy; ^11^ Otolaryngology and Audiology Unit IRCCS Azienda Ospedaliero‐Universitaria of Bologna Bologna Italy; ^12^ Department of Otolaryngology—Head and Neck Surgery IRCCS Regina Elena National Cancer Institute Rome Italy; ^13^ Department of Medical, Surgical and Health Sciences, Section of Otolaryngology University of Trieste Trieste Italy; ^14^ Department of Biomedical Sciences Humanitas University Milano Italy; ^15^ Otorhinolaryngology Unit IRCCS Humanitas Research Hospital Milan Italy; ^16^ Department of Sense Organs 'Sapienza' University of Rome Rome Italy; ^17^ Department of Otorhinolaryngology—Head and Neck Surgery IRCCS San Raffaele Scientific Institute Milan Italy; ^18^ Vita‐Salute San Raffaele University Milan Italy; ^19^ Department of Experimental and Clinical Medicine University of Florence Florence Italy; ^20^ Otorhinolaryngology Unit A. Gemelli University Hospital Foundation IRCCS Rome Italy; ^21^ Department of Head, Neck and Sensory Organs Catholic University of the Sacred Heart Rome Italy; ^22^ Department of Otorhinolaryngology—Head and Neck Surgery University Hospital Ostrava and Faculty of Medicine Ostrava Czech Republic; ^23^ Department of Head and Neck Surgery University of Sao Paulo School of Medicine Sao Paulo Brazil; ^24^ Oncology Department University of Turin Italy; ^25^ Department of Surgical and Medical Specialties, Radiological Sciences, and Public Health, Unit of Otorhinolaryngology‐Head and Neck Surgery, ASST Spedali Civili of Brescia Brescia Italy

**Keywords:** clinically negative neck, larynx, limited neck dissection, occult metastasis, supraglottis

## Abstract

**Background:**

Supraglottic squamous cell carcinoma (SCC) is a significant portion of head and neck cancers, with the management of clinically negative necks (cN0) through selective neck dissection (SND) being debated due to potential morbidities and low metastasis rates in levels IIb and IV.

**Methods:**

This study is a retrospective, multicenter examination of the potential feasibility of limited neck dissection (LND), including only levels IIa and III in cN0 supraglottic SCC patients. It analyzed occult metastasis rates and explored relapse occurrences alongside potential predictors of lymph node metastasis.

**Results:**

Among 425 patients, predominantly male (85.6%) with a mean age of 63 years, the occult metastasis rate was 28.9%, and 13.7% experienced relapses during a mean follow‐up of 52 months. Advanced clinical stage, higher grading, and other risk factors emerged as predictors of occult lymph node metastasis at level IIb.

**Conclusions:**

The study supports LND potential feasibility for cN0 supraglottic SCC, suggesting level IIb dissection can be omitted in specific early‐stage cases to reduce morbidity without affecting outcomes.

## 1 Introduction

Laryngeal squamous cell carcinoma (SCC) represents 20.8% of the SCC of head and neck and supraglottic SCC is responsible for 30%–40% of cancers affecting the larynx [1].

Lymph node metastasis is the most significant prognostic factor for SCC of the larynx and selective neck dissection (SND) of levels II–IV is recommended for patients with T1‐T4 supraglottic SCC with clinically negative neck (cN0) [2, 3]. The primary risks associated with II‐IV SND are shoulder dysfunction related to dissection maneuvers on the spinal accessory nerve at level IIb [4] and lesions of the phrenic nerve and thoracic duct at level IV [5]. Many authors have demonstrated that level IIb lymph nodes are rarely involved in metastatic disease in laryngeal SCC with cN0. The incidence of occult metastasis was reported to be 0%–2% in level IIb [6, 7, 8, 9] and 0%–3,4% in level IV [10, 11, 12].

Limited neck dissection (LND), involving only levels IIa and III, has been proposed as an alternative approach. While some studies have shown that LND of levels IIa and III is oncologically safe in selected cN0 cases of supraglottic laryngeal cancer, the reliability of these findings is constrained by limitations in sample size across studies. Our study aims to elucidate the efficacy and safety of LND in this context.

## 2 Materials and Methods

This retrospective multicenter international study involved 15 Otorhinolaryngology‐Head and Neck Surgery divisions. The study protocol was approved by the local ethics committee. The recruited centers are listed in Table 1.

**TABLE 1:** Recruited centers.

Otolaryngology‐Head and Neck Department, University of Verona, Italy
Otorhinolaryngology, Head and Neck Surgery, IRCCS Azienda Ospedaliero‐Universitaria di Bologna, Policlinico S. Orsola‐Malpighi, Bologna, Italy
Otorhinolaryngology‐Head and Neck Surgery, ASST Spedali Civili of Brescia, University of Brescia, School of Medicine, Brescia, Italy
Otolaryngology‐Head & Neck Surgery, IRCCS Regina Elena National Cancer Institute, Rome, Italy
Otorhinolaryngology Unit, IRCCS Humanitas Research Hospital, Rozzano, Italy
Otorhinolaryngology‐Head and Neck Surgery, University Hospital of Modena, Modena, Italy
Head and Neck Surgery Department, University of Sao Paulo School of Medicine, Sao Paulo, Brazil
Otorhinolaryngology, San Luigi Gonzaga Hospital, University of Turin, Turin, Italy
Otolaryngology‐Head and Neck Surgery, San Raffaele Scientific Institute, Vita‐Salute University, Milan, Italy
Unit of Otorhinolaryngology, Vittorio Veneto Hospital, Vittorio Veneto, Italy
Otolaryngology, Department of Medical, Surgical and Health Sciences, University of Trieste, Trieste, Italy
Otorhinolaryngology‐Head and Neck Surgery, IRCCS Ospedale Policlinico San Martino, Genoa, Italy
Otorhinolaryngology‐Head and Neck Surgery, University Hospital Ostrava, Ostrava, Czech Republic
Otorhinolaryngology, Fondazione Policlinico Universitario A. Gemelli IRCCS, Rome, Italy
Head and Neck Oncology and Robotic Surgery, Azienda Ospedaliero‐Universitaria Careggi

The inclusion criteria were as follows: (1) patients affected by supraglottic laryngeal SCC (all sites included); (2) cN0 documented by computed tomography (CT) or magnetic resonance imaging (MRI); (3) no distant metastasis documented by CT or MRI or positron emission tomography (PET); (4) surgical protocol of total laryngectomy (TL) or open partial horizontal laryngectomy (OPHL) any type or endoscopic supraglottic laryngectomy (ESL) any type + SND II‐IV (always including level IIa, IIb, III, and IV) ipsilateral or bilateral; (5) minimum follow‐up of 1 year, and (6) high‐quality neck dissection (ND) with a minimum lymph node yield of 18 in a single neck.

The exclusion criteria were: (1) previous radiotherapy (RT) or chemoradiotherapy (CHTRT) treatment on the neck; (2) previous surgery on the neck; and (3) previous head and neck cancer.

The following data were collected from the medical records and codified: demographic data, risk factors (alcohol consumption and smoking), treatment modalities (surgery +/− adjuvant treatment), surgical procedures (type of laryngectomy and neck dissection with involved levels), oncological clinical and pathological staging, number of metastases by level, intraoperative complications (spinal accessory nerve injuries, phrenic nerve injuries, and thoracic duct injuries), post‐operative complications (shoulder mobility impairment, dypnea, and chilous leak), and follow‐up status. The American Joint Committee on Cancer (AJCC) Staging System, 8th edition [13] was used to define oncological stage.

Stata 17.0 was used for statistical analysis. The primary outcomes were to assess the rates of occult metastases in patients with supraglottic cN0 SCC and analyze the risk factors linked to occult metastases at levels IIb and IV. The oncological outcomes and relapses were the secondary endpoints. Independent variables (age, gender, smoking habit as current or previous smoker, alcohol consumption, clinical staging, grading, type of neck dissection as monolateral or bilateral, total number of metastatic lymph nodes) were investigated with univariate and multivariate Cox proportional hazard models. A *p*‐value < 0.05 was considered as the threshold for statistical significance. The *T*‐test was used for normally distributed quantitative variables (Shapiro–Wilk test), and the Mann–Whitney test was used for non‐normally distributed quantitative variables. As regards the qualitative variables, the Chi‐square with Fisher's exact correction was used.

## 3 Results

### 3.1 Clinicopathological Characteristics

Overall, 425 patients (mean age 63.0 years, range: 31–90 years), of whom 364 (85.6%) were men, met the inclusion criteria. All patients were staged as cN0. The mean follow‐up duration was 52 months (range: 12–201 months). 59% of the patients included achieved 3 years of follow‐up, while only 12% of the total patients exceeded 5 years of follow‐up. Table 2 summarizes the main features of the recruited patients.

**TABLE 2:** Characteristics of the recruited patients.

Variables	Number of patients OR %
Median age (years)	63.0 (9.5%)
Gender	
F	61 (14.4%)
M	364 (85.6%)
Smoke	
No	8.4%
Yes	75.1%
Ex	16.5%
Alcoholism	
No	77.5%
Yes	21.2%
Ex	1.3%
cTNM	
cT1N0M0	16 (3.8%)
cT2N0M0	111 (25.9%)
cT3N0M0	165 (38.9%)
cT4aN0M0	133 (31.4%)
cTNM binary	
cT1N0M0/cT2N0M0	127 (29.7%)
cT3N0M0/cT4aN0M0	298 (70.3%)
Grading	
G 1	18 (3.8%)
G 2	258 (61.3%)
G 3	149 (34.9%)
Neck dissection	
Monolateral	81 (19.1%)
Bilateral	344 (80.9%)

All patients underwent SND, including levels IIa, IIb, III, and IV. The ipsilateral SND was performed for patients with laterally located lesions not reaching the midline. A bilateral neck dissection was performed if the supraglottic SCC was reaching and crossed the midline.

### 3.2 Incidence of Lymph Node Metastasis

The average number of lymph nodes harvested was 21.4 for unilateral neck dissections and 45.7 for bilateral neck dissections. Among 302 patients, no lymph node metastases were detected (71.1%), while in 123 patients, at least one metastatic lymph node was identified (28.9%).

Out of all the patients included, occult metastases were individually detected at the following levels: in 57 cases (13.4%) at level IIa, in 18 cases (4.2%) at level IIb, in 67 cases (15.8%) at level III, and in 26 patients (6.1%) at level IV.

When examining synchronous metastatic involvement of sublevel IIb and other levels in our series, we observed simultaneous metastases at level IIa in one case, at level III in nine cases, and at level IV in two cases. None of these associations were found to be statistically significant.

When considering synchronous metastatic involvement of sublevel IV and other levels in our series, there were simultaneously metastases at level IIa in 9 cases, at level IIb in 2 cases, and at level III in 21 cases. None of these associations were found to be statistically significant. Extranodal extension (ENE) in general was observed in 21 patients (4.9%).

Table 3 summarizes these data.

**TABLE 3:** General characteristics and distribution of lymph node metastasis.

Variable	*p*	Number OR %
Average lymph nodes harvested (unilateral)	—	21.4
Average lymph nodes harvested (bilateral)	—	45.7
Patients with no lymph node metastases	—	302 (71.1%)
Patients with at least one metastatic lymph node	—	123 (28.9%)
Level IIa occult metastasis	—	57 cases (13.4%)
Level IIb occult metastasis	—	18 cases (4.2%)
Level III occult metastasis	—	67 cases (15.8%)
Level IV occult metastasis	—	26 cases (6.1%)
Synchronous metastasis (IIb and IIa)	> 0.05	1 case
Synchronous metastasis (IIb and III)	> 0.05	9 cases
Synchronous metastasis (IIb and IV)	> 0.05	2 cases
Synchronous metastasis (IV and IIa)	> 0.05	9 cases
Synchronous metastasis (IV and IIb)	> 0.05	2 cases
Synchronous metastasis (IV and III)	> 0.05	21 cases
Extranodal extension (ENE)	—	21 patients (4.9%)

The risk of occult metastases was higher for cT3‐cT4 stages than for cT1‐cT2 stages (*p* = 0.0001, OR 2.79, 95% C.I. 1.62–4.82). Furthermore, the risk of death was higher in patients with occult lymph node metastases (*p* = 0.004, HR 2.01, 95% C.I. 1.3–3.2).

Table 4 summarizes the explained data.

**TABLE 4:** Risk of occult metastasis and risk of death.

Variable	Comparison	*p*	OR/HR	95% C.I.
Risk of occult metastasis	cT3‐cT4 vs. cT1‐cT2	0.0001	OR 2.79	1.62–4.82
Risk of death	Patients with occult lymph node metastases vs. those without	0.004	HR 2.01	1.3–3.2

### 3.3 Predictors of Level IIB and IV Nodal Metastasis

To investigate risk factors for level IIb lymph node metastasis, eight variables (age, gender, smoking habit as current or previous smoker, alcohol consumption, clinical staging, grading, type of neck dissection as unilateral or bilateral, and total number of metastatic lymph nodes) were included in a univariate analysis.

Age > 61, smoking habit, alcohol consumption, advanced clinical staging (cT3‐cT4), grading (G2‐G3), and type of neck dissection (intended as bilateral) were all associated with the presence of occult metastases at level IIb (*p* < 0.05).

The same approach was undertaken to investigate potential risk factors for occult metastasis at level IV. Occult metastases at level IV were found to be statistically associated with a higher total number of metastatic lymph nodes (*p* = 0.0001).

In the multivariate analysis, the variables studied did not achieve statistical significance as predictors of level IIb and IV lymph node metastasis.

Table 5 summarizes the potential predictors of level IIb and IV nodal metastasis.

**TABLE 5:** Association of variables with level IIb and IV metastasis.

Variables	Level IIb *p*	Level IV *p*
Age	< 0.05	0.1
Gender	0.3	0.5
Smoking habit	< 0.05	0.1
Alcohol consumption	< 0.05	0.3
Clinical staging	< 0.05	0.1
Grading	< 0.05	0.5
Type of neck dissection	< 0.05	0.1
Total number of metastatic lymph nodes	< 0.05	< 0.05

### 3.4 Complications

In terms of intraoperative complications, seven lesions of the spinal accessory nerve and two lesions of the thoracic duct were reported. Regarding postoperative complications, shoulder motility alteration was reported in 14 cases, dyspnea in nine and only one case experienced chylous leak. None of these complications were found to be statistically significant. These data are summarized in Table 6. The percentages in the table are calculated per dissection rather than per patient, as individuals with bilateral dissections face twice the risk of complications.

**TABLE 6:** Complications' description.

Complication	Number of dissections OR %		Phase
Spinal accessory nerve lesion	7	0.9%	Intraoperative
Thoracic duct lesion	2	0.3%	Intraoperative
Shoulder motility impairment	14	2%	Postoperative
Dyspnea	9	1.2%	Postoperative
Chylous leak	1	0.1%	Postoperative

### 3.5 Relapses

During follow‐up, whose median duration was 32 months, 33 (10.9%) out of 302 patients without occult lymph node metastases had relapses. Among all the 58 patients who experienced relapses (13.7%), 14 had local relapses, 24 loco‐regional relapses, 19 regional relapses, and 1 distant relapse.

In cases of loco‐regional and regional relapses, level IIb was found to be involved in only one case contralaterally to the ND performed (cT2N0M0, upstaged to pT3N2bM0, IIa, and III levels involved), while level IV was never involved. However, the patient with level IIb recurrence was treated and there was no evidence of disease at the last follow‐up.

These data are summarized in Table 7.

**TABLE 7:** Relapses rates.

Relapses	No	367 (86.3%)
	Yes	58 (13.7%)
Type of relapses	Local	14 (3.2%)
	Loco‐regional	24 (5.6%)
	Regional	19 (4.4%)
	Distant	1 (0.2)

## 4 Discussion

Lymph node metastases are the main prognostic factor in patients with supraglottic SCC. The presence of lymph node metastases appears to decrease survival in percentages ranging from 20% to 50%, depending on the clinical experience [14]. Neck dissection is a therapeutic procedure that is not free from sequelae that can impact patients' quality of life. Shoulder motility dysfunction is a significant morbidity commonly linked with ND, marked by symptoms such as shoulder droop, protrusion of the scapula, discomfort, and weakness [15]. Even when the spinal accessory nerve is anatomically preserved, normal shoulder function is not always guaranteed. Indeed, only about 70%–75% of patients who have undergone nerve‐sparing surgical procedures report being free from any shoulder‐related impairments [16, 17]. When considering the morbidity associated with ND at level IV, complications can arise, including occasional damage to the deep cervical plexus and the phrenic nerve. Chylous fistula, although rare, is another potential complication. The occurrence of such complications has been reported in studies by Lim et al. [11], Erisen, Coskun, and Basu [18], and Jong and Manni [19], with rates ranging between 5.5% and 5.8% for chylous fistulas and 2.7%–8.0% for phrenic nerve paralysis.

However, in our patient pool, the complication rates were lower, but this could also be due to the retrospective nature of this study.

Few works in the literature deal with the involvement of levels IIb and IV in supraglottic SCCs.

The first papers on the prevalence of metastases in level IIb were carried out by Elsheikh et al. [8], Jia et al. [6], Koybasioglu et al. [7], Rinaldo et al. [9], and Lim et al. [20], spanning from 2002 to 2013. These studies consistently reported a low rate of metastatic involvement in level IIb nodes, particularly in cases of laryngeal SCC without neck metastasis, with rates ranging between 0% and 2%.

More specifically, Koybasioglu et al. [7] in 2002 found no metastases in the level IIb lymph nodes across all 49 NDs. In a more extensive review, Ferlito, Silver, and Rinaldo [10] 2008 drew on data from seven comprehensive studies encompassing pathological and molecular analyses of ND specimens. In their review of 272 patients with cN0 laryngeal SCC, the incidence of metastasis in level IIb nodes was 1.4%. Furthermore, Zohdi et al. examined 14 cN0 supraglottic SCCs cases. This research found no incidence of occult neck metastases in level IIb among the subjects. Based on these findings, dissecting level IIb during ND may not be necessary for cN0 patients [21].

Regarding the metastasis rates for level IV lymph nodes, the percentages can slightly vary, generally between 0% and 3.4%, as noted by Lim et al. [11], the Brazilian Head and Neck Cancer Study Group in 1999 [12], Ferlito, Silver, and Rinaldo in 2008 [10], and Cagli, Yüce, and Güney in 2007 [22]. In contrast, Deganello et al. [23] reported the incidence of level IV lymph node involvement to be 9.5% in 122 ND carried out for 96 patients affected by cN0 laryngeal SCC.

Recent literature has begun challenging the routine practice of level IIb and IV dissection. Consequently, a trend towards more conservative ND approaches has emerged, with some institutions opting to remove lymph nodes only at levels II, or at levels II and III. This shift toward less extensive dissections is supported by studies and practices from various institutes and researchers [24, 25, 26].

To our knowledge, our study boasts the largest sample size. It provides a comprehensive analysis of clinicopathological characteristics and predictors of lymph node metastasis at levels IIb and IV in patients with supraglottic SCC undergoing SND.

Our cohort's overall incidence of lymph node metastasis was 28.9%, aligning with previous studies indicating a variable range of nodal involvement in cN0 supraglottic SCC patients. Our analysis revealed several potential predictors of lymph node metastasis, notably the stage of the disease and certain lifestyle factors such as smoking and alcohol consumption. The association between higher clinical stages (cT3‐cT4) and increased risk of occult metastases, especially at level IIb, is consistent with the literature, emphasizing the aggressive nature of advanced‐stage tumors.

In our investigation, we observed that the incidence of occult metastasis at level IIb was 4.2%, and at level IV, it was 6.1%. Notably, in univariate analysis, the presence of occult metastases at level IIb correlated with advanced stages, higher grading, age, smoking, and alcohol consumption. Conversely, for level IV, we did not identify any pre‐surgical significant associations that could inform the surgical decision regarding the type of ND to perform.

However, these findings lack confirmation through multivariate analysis, which is crucial for establishing a reliable predictive model to determine whether cN0 patients can safely forego dissection at these levels.

The findings of this study suggest that LND omitting level IIb for cN0 supraglottic SCC could be oncologically feasible, particularly for early‐stage cases. However, we recommend caution when applying these findings broadly. Level IIb should generally be dissected unless the patient presents with highly favorable factors such as early‐stage (cT1‐cT2) disease, low‐grade tumors and an absence of other high‐risk factors.

Future research should explore the development of a stratification model to better identify patients at low risk for metastases at levels IIb and IV. By doing so, the criteria for safely omitting these levels from dissection can be refined and validated in prospective studies.

### 4.1 Limitations

This study's retrospective and multicentric design introduces inherent challenges, particularly in ensuring the consistent and accurate reporting of complications. Differences in documentation practices across participating centers may lead to underreporting or inconsistencies in recorded complication rates. This is a significant limitation when interpreting this cohort's relatively low complication rates, as the actual incidence may be higher than reported.

The multicenter nature of the study also limits the ability to discern whether lymph nodes were identified intraoperatively by surgeons or postoperatively by pathologists based on pre‐established criteria. This introduces a potential bias that could affect the accuracy of the reported number of lymph nodes at each level and the rate of occult metastases.

Additionally, the study could not capture detailed data on the specific subsites of the supraglottic tumors, a limitation mainly attributable to its multicentric approach. The absence of such granularity restricts the analysis of tumor subsites and their relationship with lymph node dissection outcomes. Addressing these limitations in a standardized, prospective study could yield valuable insights.

Finally, given the methodological constraints, these findings must be interpreted with caution. Future prospective studies are necessary to comprehensively evaluate the oncological safety, functional outcomes, and optimal patient selection for LND in cN0 supraglottic SCC.

## 5 Conclusions

Despite its limitations, this research sheds light on the potential oncological safety of LND for early‐stage cN0 supraglottic SCCs. Considering the evidence gathered it may appear judicious to forego level IIb dissection in specific instances.

## Author Contributions

Conceptualization: Sara Bassani. Methodology: Giuditta Mannelli and Sara Bassani. Writing – original draft preparation: Sara Bassani, Giuditta Mannelli, and Filippo Marani performed the statistical analysis. All the other authors contributed equally to collecting data and reviewing the original draft. All authors have read and agreed to the published version of the manuscript.

## Ethics Statement

This study protocol was reviewed and approved by Comitato etico per la sperimentazione clinica of A.O. Universitaria Integrata of Verona, University of Verona, Italy, approval number prog. 3651cesc.

## Consent

The authors have nothing to report.

## Conflicts of Interest

The authors declare no conflicts of interest.

## Data Availability

The data that support the findings of this study are available from the corresponding author upon reasonable request.
